# Cutaneous Eruption in a Crohn’s Disease Patient Under Tumor Necrosis Alpha Inhibitor Treatment: A Rare Case of Syphilis

**DOI:** 10.7759/cureus.57003

**Published:** 2024-03-26

**Authors:** Georgia Bellou, Eirini Zaharopoulou, Anastasios Giannoukos, Olga Kosmopoulou, Maria Tzouvala

**Affiliations:** 1 Gastroenterology, General Hospital of Nikea and West Attica, Athens, GRC; 2 Dermatology, Andreas Syngros Hospital of Venereal & Dermatological Diseases, Athens, GRC; 3 Internal Medicine, General Hospital of Nikea and West Attica, Athens, GRC

**Keywords:** syphilis, cutaneous eruptions, extra-intestinal manifestations (eims), tumor necrosis factor-α inhibitors (anti-tnfα), crohn’s disease (cd)

## Abstract

Reaching a diagnosis of a cutaneous eruption in a Crohn’s disease (CD) patient treated with anti-tumor necrosis factor alpha (anti-TNFα) can be challenging. Differential diagnosis must include extra-intestinal manifestations of CD, adverse reactions to the therapy itself as well as infectious diseases with cutaneous manifestations. We report the case of a 28-year-old man on infliximab for Crohn’s colitis, who presented with painless, non-pruritic genital and body exanthema. After a thorough evaluation, early secondary syphilis was confirmed with a fluorescent treponemal antibodies-absorbed test. Intramuscular (IM) benzathine penicillin G 2.4 million units in a single dose was administered and clinical manifestations resolved completely within a couple of weeks.

## Introduction

Crohn’s disease is an idiopathic, chronic inflammatory bowel disease (IBD) affecting the gastrointestinal tract. It is also characterized by extra-intestinal manifestations (EIMs) which may include the skin in 2-34% of IBD patients. The most common skin EIMs associated with CD are pyoderma gangrenosum and erythema nodosum. Other rare cutaneous EIMs include metastatic CD affecting the perineum, feet, legs, penis and vulva, leukocytoclastic vasculitis, Sweet syndrome, cutaneous polyarteritis nodosa, and epidermolysis bullosa acquisita. Furthermore, increased occurrence of psoriasis has been described among patients with CD, although it is not considered an EIM of the disease itself. Treatment for CD includes immunosuppressive medications, such as biologics and novel small molecules. A commonly used biologic agent is an anti-tumor necrosis factor alpha (anti-TNFα) monoclonal antibody which is associated with cutaneous adverse events such as psoriasiform rash or eczema and opportunistic dermatologic infections as well such as pityriasis rosea, dermatophytes, herpes, and *Staphylococcus aureus* [1-3].

Thus, a CD patient treated with anti-TNFα agents who presents with a cutaneous eruption requires a thorough clinical approach and syphilis should always be included in the differential diagnosis as it is considered “The Great Imitator.”

Syphilis is an infectious disease caused by the spirochete *Treponema pallidum* and is primarily transmitted sexually by contact with the infectious lesions, as well as vertically from mother to fetus and via blood and blood products. The disease is characterized by three stages manifesting with clinical symptoms, primary, secondary, and tertiary syphilis, with latent asymptomatic stages in between. Primary syphilis typically manifests with a painless and highly contagious ulcer at the site of insertion, usually occurring on the external genitalia, perineum, and anus, and known as chancre. Secondary syphilis appears six to 12 weeks after the primary infection when the spirochete spreads systemically through the bloodstream. Clinical manifestations of the secondary stage may include non-specific symptoms such as fever, fatigue, generalized lymphadenopathy, as well as syphilitic dermatitis and condylomata lata which are characteristic of this stage. If left untreated, tertiary syphilis may develop and involvement of the cardiovascular system, central nervous system, and other internal organs occurs [4,5]. It has been reported that in patients on immunosuppressive therapy, syphilis may present with an accelerated clinical course and develop rapidly tertiary syphilis [6,7].

## Case presentation

**Figure 1:**
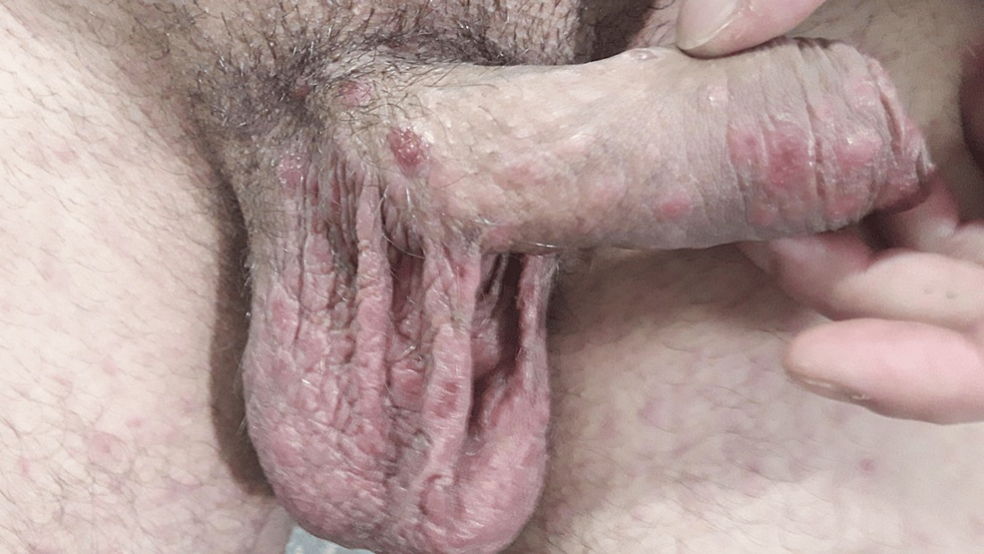
Salmon-colored lesions on external genitalia Image credit: Georgia Bellou

**Figure 2:**
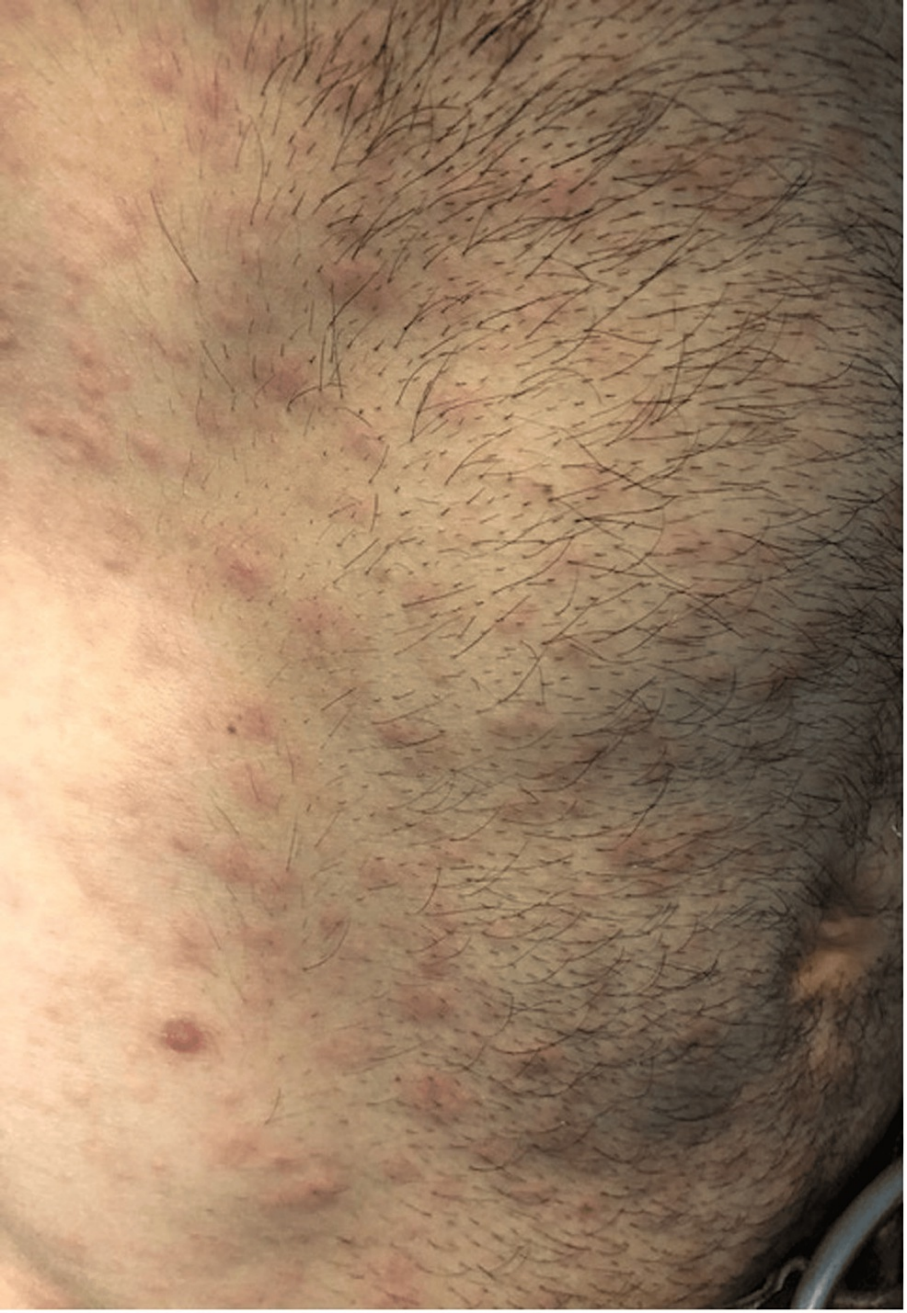
Salmon-colored lesions on the trunk Image credit: Georgia Bellou

**Figure 3:**
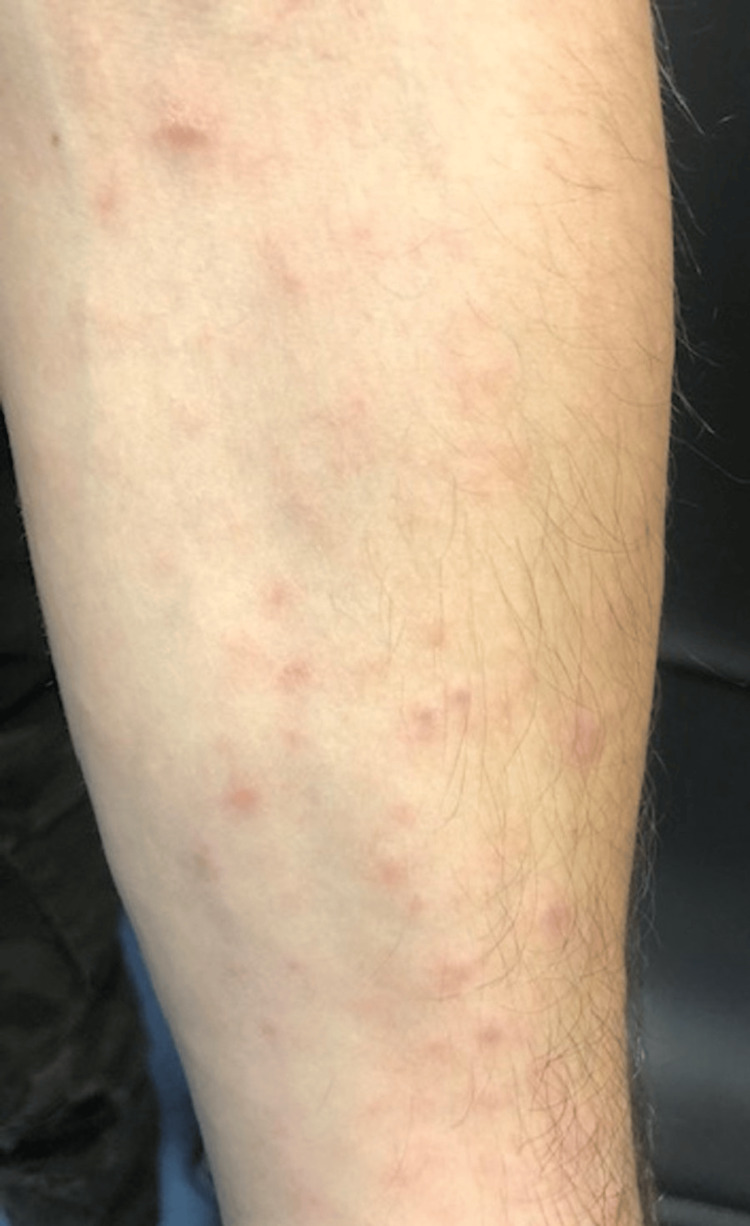
Salmon-colored lesions on the limbs Image credit: Georgia Bellou

We herein present a case of a 28-year-old male patient with CD treated with anti-TNFα immunosuppressive therapy (Infliximab 5 mg/kg every eight weeks), for six years, in clinical and endoscopic remission, presented with a sudden cutaneous eruption of salmon-colored non-pruritic lesions. The rash appeared in the beginning on his external genitalia (Figure 1) seven weeks after the last infliximab infusion and expanded on the trunk (Figure 2) upper and lower limbs, (Figure 3), including the palms and soles, and was accompanied by mild diffuse arthralgia and elevated inflammatory markers (WBC: 10.830, ESR: 73 mm/h, CRP: 92.5 mg/l {upper normal limit 3 mg/l}).

Infliximab infusion was temporarily withheld. After the initial dermatologic consultation, we performed viral assays including antibodies for Epstein-Barr, Cytomegalovirus, Coxsackie, Echo, and HIV, as well as microbiological and immunological assays including antibodies for Toxoplasma, throat strep test for detection of streptococcal group A antigen, lupus anticoagulant, anti-cardiolipin antibodies and venereal disease research laboratory test for detection of *Treponema pallidum* antibodies which were all negative.

The type and the distribution of the rash, combined with the gradual clinical deterioration of the patient with fatigue and arthralgia raised again the suspicion of early secondary syphilis which was eventually confirmed with a fluorescent treponemal antibodies-absorbed test.

Following diagnosis, we thoroughly interviewed the patient, who could not recall a primary syphilitic chancre, but he admitted frequent unprotected sexual intercourse. Intramuscular (IM) benzathine penicillin G 2.4 million units in a single dose was administered and clinical manifestations resolved completely within a couple of weeks. Three weeks post-treatment, inflammatory markers returned to normal and infliximab infusion was resumed without relapse of the infection after an 18-month follow-up.

## Discussion

A cutaneous eruption in a Crohn’s disease patient under anti-TNFα therapy can be challenging. Differential diagnosis must include extra-intestinal manifestations of Crohn’s disease, adverse reactions to the therapy itself, as well as infectious diseases with cutaneous manifestations. In such cases, medical history is of great importance, as well as the fact that this group of patients may exhibit an accelerated clinical course when infected due to immunosuppressive therapy.

Our patient presented with a generalized maculopapular rash, affecting the palms and soles, accompanied by non-specific symptoms, and elevated inflammatory markers, and manifested seven weeks after infliximab infusion. The patient was in clinical and endoscopic remission, thus disease exacerbation and EIMs associated with it were ruled out. The type and the distribution of the rash helped to narrow down the differential diagnosis and exclude other EIMs and drug-induced adverse reactions. Furthermore, patients treated with biologics are immunosuppressed, and thus the risk of infections is higher in such individuals. Despite the fact that non-treponemal tests were negative, the clinical picture raised high suspicion of early secondary syphilis which was eventually confirmed by fluorescent treponemal antibodies-absorbed test.

In our literature review using PubMed, we found only six cases of syphilis under anti-TNFα and they were mainly treated with intravenous penicillin even in the absence of neurologic symptoms (Table 1) [2,6-10].

**Table 1:** Cases of syphilis in patients under anti-TNFα and their treatment described in the literature (PubMed) IM, intramuscular; IU, International Units; IV, intravenous; PCr, polymerase chain reaction; PO, per os; anti-TNFα, anti-tumor necrosis factor alpha

Reference	Disease	Drug	Neurological manifestation	Treatment
Bettenworth et al. [6]	Ulcerative colitis	Infliximab	No	Penicillin 10 million IV IU 14 days
Kase et al. [7]	Psoriasis vulgaris	Infliximab	Yes (emotional lability and excitability)	Penicillin 24 million IV IU 10 days and ceftriaxone 28 days
Asahina et al. [8]	Rheumatoid arthritis	Initially, adalimumab but changed to infliximab on suspicion of paradoxical reaction	No	Amoxicillin (no dose or treatment duration specified)
Bories-Haffner et al. [2]	Ankylosing spondylitis	Infliximab	No	Penicillin 24 million of IV IU 15 days
Assikar et al. [10]	Ankylosing spondylitis	Etanercept	Yes (hypomanic episode)	IV penicillin 14 days (no dose specified)
Iglesias Plaza et al. [9]	Ankylosing spondylitis	Golimumab	No	Penicillin G 2.4 million IM total 3 doses and due to positive pharyngeal PCR for Neisseria gonorrhoeae ceftriaxone 250 mg IV and azithromycin 1 g PO

However, treatment guidelines for primary and secondary syphilis recommend benzathine penicillin G 2.4 million units intramuscularly in a single dose among the general population and persons with HIV infection. Treatment guidelines for immunosuppressed individuals or patients under biologics are not available. We administered our patient the recommended regimen, with excellent response and no relapse after long follow-up [11].

## Conclusions

In conclusion, the clinical approach to a Crohn’s disease patient with cutaneous lesions under anti-TNFα treatment is complex. Acquisition of a thorough medical history is critical for reaching the correct diagnosis early, initiate appropriate treatment and avoid serious complications in immunocompromised patients. Diagnostic work-up should include infectious diseases, such as syphilis, when clinical course is suspicious. Hence, an interdisciplinary approach is fundamental. Treatment options for syphilis in patients on biologic therapy induce skepticism, since there are no specific guidelines for such cases, so far.
